# How challenge stressors affect deviant innovation behavior among Chinese frontline textile workers in the age of artificial intelligence

**DOI:** 10.1038/s41598-025-11428-6

**Published:** 2025-07-18

**Authors:** Xiaoru Du, Chunzi Cai

**Affiliations:** School of Art and Design, Wuhan Textile University, Wuhan, China

**Keywords:** Challenge stressors, Deviant innovation behavior, Psychological empowerment, Satisfaction with human–robot collaboration, Frontline mill workers, Psychology, Human behaviour

## Abstract

Previous research has examined the impact of internal organizational stress on employee innovation behavior, but recent research has not thoroughly examined whether artificial intelligence can aid frontline workers in innovating beyond their workflow. This study explores the impact of challenge stressors on deviant innovation behavior among employees of a textile mill based on the conservation of resources theory. This empirical study surveyed a sample of 400 frontline employees of a textile mill in China. Results revealed that challenge stressors significantly positively impact employees’ deviant innovation behavior, and that psychological empowerment mediates their relationship. In addition, satisfaction with human–robot collaboration moderates the relationship between challenge stressors and deviant innovation behavior. Higher satisfaction can enhance employees’ willingness to engage in innovative behaviors, even in the presence of challenge stressors, while lower satisfaction may hinder such behaviors. These findings offer new insights into how stressors affect employees’ deviant innovation behavior and provide valuable guidance for human resource management practices.

## Introduction

Amid fierce global economic competition, the textile industry, a crucial part of traditional manufacturing, faces increasing pressure to transform and upgrade^1^. This pressure arises from factors such as changing consumer demands, rapid technological advancements, fluctuating raw material prices, rising labor costs, and trade uncertainties^2^. These challenges compel textile companies to innovate continuously to remain competitive. While artificial intelligence (AI) has improved production efficiency and product quality^3^, frontline employees are stressed by balancing production tasks and adapting to new technologies^4–7^. These employees are key to identifying production issues and proposing process improvements^8^, but their ability to innovate is limited by high workloads and lack of organizational support^9,10^. Therefore, understanding how these pressures, particularly challenge stressors, influence innovation behavior in frontline workers is essential.

Innovation is a critical driver of business success in today’s competitive environment. Organizations depend on employees to generate ideas that optimize production processes and improve product quality^11^. However, innovation is shaped by various work-related factors, especially stress. Stressors can be classified into hindrance and challenge stressors^12^. Hindrance stressors, such as role conflict and job insecurity, hinder progress and personal growth. In contrast, challenge stressors, like increased workload and time pressure, are viewed as opportunities for growth and can unlock employees’ potential^13^. While these stressors can boost motivation and creativity^14^, if not managed well, they can lead to burnout^15^. Previous research has primarily focused on general innovation behavior^16^, overlooking the influence of challenge stressors on deviant innovation behavior, which involves breaking norms to solve problems.

This study addresses this gap by examining how challenge stressors impact deviant innovation behavior in frontline workers in the textile industry. Deviant innovation behavior often arises under stress and can lead to creative problem-solving when employees deviate from standard procedures^17^. This research contributes to understanding how challenge stressors affect workplace creativity, particularly during technological transformations in traditional industries. Building on the Conservation of Resources (COR) theory, which suggests individuals are motivated to protect and gain valuable resources^18^, this study also explores the indirect roles of psychological empowerment and satisfaction with human–robot collaboration in the relationship between challenge stressors and deviant innovation behavior. The findings will offer practical insights for how textile companies can use challenge stressors to foster innovation while maintaining employee well-being.

### Challenge stressors and deviant innovation behavior

Deviant innovation behavior refers to spontaneous and covert innovative actions carried out by an individual without formal recognition from the organization^18^. In contrast to innovative work behavior, DIB often involves breaking existing norms and processes, using unconventional approaches to solve problems^18^. Innovative work behavior, on the other hand, typically involves formal and publicly recognized innovative activities that align with organizational standards and expectations^18^. The innovation process requires significant investment of personal resources such as time, energy, and attention, which are crucial for innovation^18^. The Conservation of Resources (COR) theory suggests that individuals’ behaviors are primarily driven by the need to acquire, maintain, and protect their resources^19^. This theory emphasizes that individuals continually strive to obtain, preserve, and safeguard valuable resources, such as time, energy, attention, and emotional support^19^. These resources play a crucial role in helping individuals cope with challenges, complete tasks, and foster innovation^19^. In stressful situations, the protection and depletion of resources directly affect individuals’ mental health, work performance, and innovative abilities^19^. Workplace stressors can impact employees differently depending on their nature^17,20^. While challenge stressors can act as a catalyst for innovation by motivating employees to overcome obstacles, hindrance stressors tend to deplete resources and obstruct innovation^17,20^. Thus, stress in organizational life is a double-edged sword, with its effects varying based on the type of stressor^17,20^. Challenge stressors, as positive job demands, have been shown to stimulate employees’ intrinsic motivation, enhancing their autonomy, competence, and engagement, which in turn fosters innovative ideas and personal growth^21–23^. Moreover, challenge stressors increase employees’ interest and sense of meaning in their work, driving innovative behavior^24,25^.

As a unique form of innovation beyond organizational processes, deviant innovation behavior is often kept secret due to its high risk, but employees will disclose it to the organization once sufficient evidence supports its feasibility and value^18^. Challenge stressors, as job demands that boost resources, can stimulate employees’ intrinsic motivation, enhancing autonomy, competence, and initiative, thus fostering innovation^22,26^. In contrast, if employees experience resource loss when facing hindrance stressors, such as time pressure, lack of organizational support, they may experience burnout, which will reduce their innovative aptitude^17,27^. Consequently, we predict that challenge stressors, by stimulating intrinsic motivation and innovative potential, promote employees’ deviant innovation behaviors. Based on the above theories and reasoning, we propose the Hypothesis 1: Challenge stressors positively affect deviant innovation behavior.

### Mediating role of psychological empowerment

Based on the COR theory, individuals’ behaviors are driven by the need to acquire, maintain, and protect their resources^19^. Within this theoretical framework, challenge stressors are viewed as a positive work demand that can stimulate employees’ intrinsic motivation, thereby indirectly influencing their deviant innovation behavior. Specifically, challenge stressors enhance employees’ psychological empowerment, which in turn impacts their deviant innovation behavior. Regarding how challenging stressors affect deviant innovation, psychological empowerment might be an underlying factor at play^23,28,29^. Psychological empowerment emphasizes the subjective experience of individuals at work, encompassing psychological processes such as self-determination, competence, self-efficacy, and the meaning of work, it stresses employees’ perceptions of autonomy and influence at work^23,28,29^. Cognitive appraisals of stress revealed individual stress results from interactions between individuals and their environments. Individuals can identify and differentiate different stressor types and adopt appropriate coping strategies to manage work stress, optimizing their psychological cognition and achieving their goal^30^. Challenge stressors are commonly viewed as opportunities for career development. When faced with new tasks or high-demand work, employees initially evaluate the relevance of the task to their personal goals (primary appraisal), potential benefits, and challenges to their abilities. And then, they will perform a secondary assessment (secondary appraisal) to assess their abilities and resources to handle the demands of the task, if they believe they can meet the challenge, they will feel more confident and have a sense of control, thus exhibiting a stronger sense of self-determination and competence (two primary manifestations of psychological empowerment). This is helpful to employees adopt positive coping strategies to overcome challenges, promoting the formation of new ideas and personal growth. Given this, challenge stressors can influence psychological empowerment by affecting an individual’s cognitive evaluation process.

Psychological empowerment is one of the factors that affect an individual’s intrinsic motivation. Those who have a high level of psychological empowerment are more likely to have strong intrinsic motivation^29,31^. The self-determination theory posits that individuals have both autonomous and controlled motivations. Individuals possess an inherent need for competence and autonomy, which potentially motivates them to leverage their skills to propose innovative ideas, expand their job responsibilities, and engage in extra-role behaviors^26,32^. Providing support and resources can stimulate employees’ intrinsic motivation, which in turn promotes deviant innovation behavior. Thus, intrinsic motivation can drive highly psychologically empowered employees to maintain enthusiasm for work. Employees with high self‐determination, strong work motivation, and robust internal drive exhibit greater confidence in tackling high-risk work and resource scarcity; they are convinced of their ability to overcome challenges and invest further to actualize their self-worth^32^. Given this, psychological empowerment can stimulate deviant innovation behavior among employees by influencing their intrinsic motivation. Previous studies have shown that challenge stressors can positively impact employees’ psychology, specifically influencing psychological empowerment^15^. Specifically, employees who view challenges as opportunities for self-improvement tend to exhibit higher proactivity, the greater an employee’s competence, the more capable they feel in completing work tasks^26^. Furthermore, employees’ engagement increases when they perceive their work as valuable, leading them to actively seek solutions and enhance their sense of autonomy^26^. Existing literature has shown that a positive leadership style can increase employees’ psychological safety, autonomy, competence, and sense of meaning in their work, promoting the development of deviant innovation behavior^33^. Therefore, based on the cognitive appraisal theory of stress and self-determination theory, this paper proposes the Hypothesis 2: Psychological empowerment mediates the relationship between challenge stressors and deviant innovation behavior.

### Moderating role of satisfaction with human–robot collaboration

Beyond the effects of psychological empowerment, satisfaction with human–robot collaboration (SHRC) is a significant determinant. The COR theory not only suggests that individuals’ behaviors are primarily driven by the acquisition, maintenance, and protection of resources, but also proposes that employees’ resource needs and resource depletion are influenced by other factors in the work environment^19^. SHRC, as an important moderating variable, may alter employees’ cognitive appraisal processes and moderate the impact of challenge stressors on deviant innovation behavior. Specifically, high SHRC can enhance employees’ sense of trust and control, providing the necessary psychological support for them to maintain high innovation motivation when facing challenge stressors. In contrast, low SHRC may lead to emotional instability and job insecurity, thereby weakening the positive effects of challenge stressors. Relevant research has found that techno-invasion can affect employees’ deviant innovation behavior^34^. Whether high and low levels of SHRC differentially impact the effect of challenge stressors on deviant innovation behavior merits further exploration. The situation awareness theory suggests that effective human–robot collaboration depends on the user’s accurate understanding of the system status and environment^35^. The more employees clearly understand an AI system, the more likely they are to collaborate effectively with these systems, improving job satisfaction and performance. A survey conducted by *MIT Sloan Management Review* and Boston Consulting Group showed that nearly 70% of respondents were not worried about AI automating their work tasks. In contrast, they hoped that AI could take over some tedious and mechanized tasks, allowing them to acquire new skills while enhancing existing ones^36,37^. SHRC, a vital indicator of the quality of interaction between workers and AI, significantly influences the effectiveness and value of this cooperative relationship^38^.

**Fig. 1:**
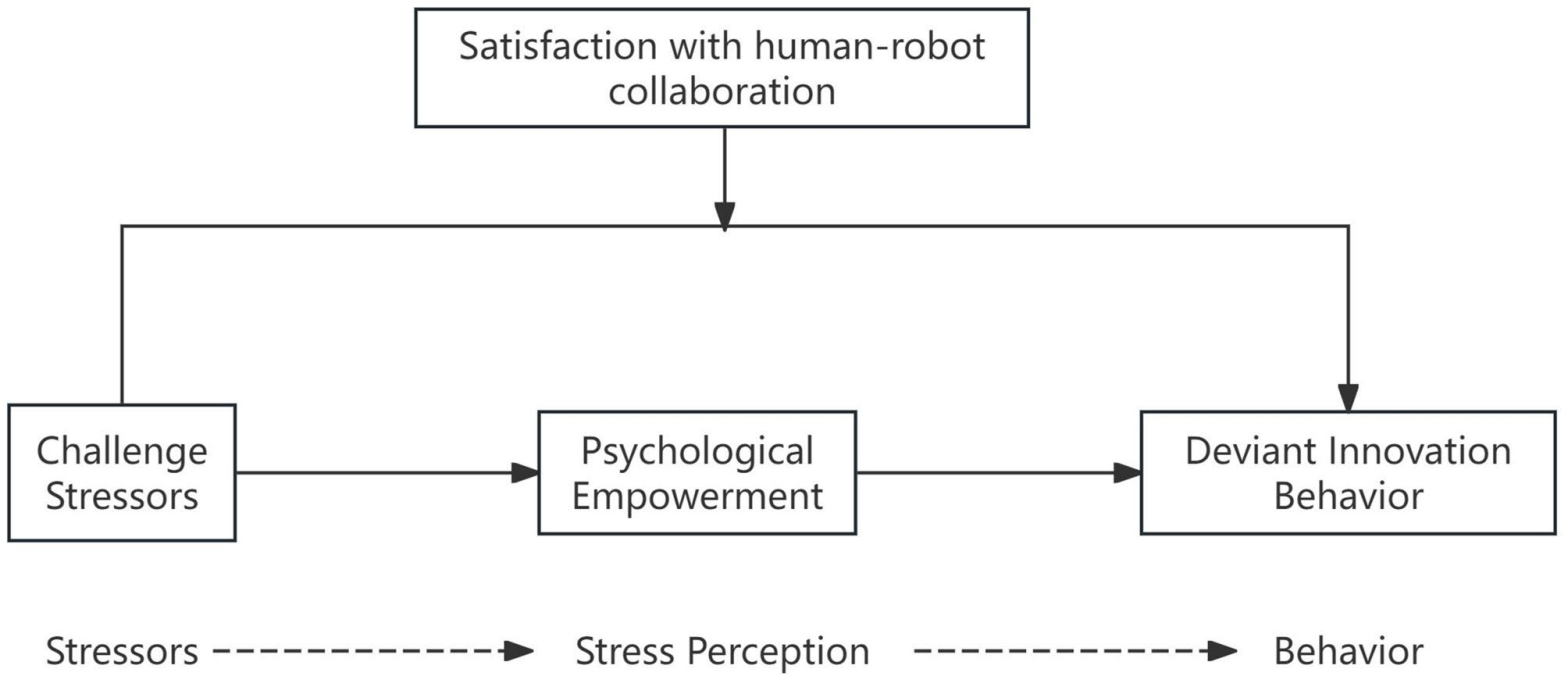
Hypothetical model.

Satisfaction, trust, and commitment constitute the three cornerstones of a successful cooperative relationship^39^, with satisfaction representing the direct feedback of workers regarding their experience of collaborating with AI. Under high-intensity task pressures, employees with higher trust in and commitment to the AI system are more likely to rely on it for innovation and learning when facing challenge stressors^40^. In addition, an environment with high SHRC can provide a sense of psychological comfort and support, increasing employees’ willingness to engage in high autonomy and high-risk innovation behaviors^41^. However, if employees are skeptical about collaborating with AI and worry about being replaced by AI technology, the resulting job insecurity will negatively affect their emotional state, potentially hindering their attempts at innovation^42^. Given this, high SHRC enhances the positive impact of challenge stressors on innovation behavior, whereas low satisfaction attenuates this effect. This study suggests that SHRC is an important moderating variable, proposing the Hypothesis 3: Satisfaction with human–robot collaboration moderates the impact of challenge stressors on deviant innovation behavior. And Fig. 1 shows the hypothesized model constructed based on previous theories and studies.

## Method

### Ethical approval

The study was approved by the Wuhan Textile University (approval code: WTU-240111-001; approval date: 18 January 2024). The questionnaire survey on employees’ psychological issues is based on the principles of voluntariness and informed consent, and it maximizes the protection of employees’ rights and privacy. All methods adhered to relevant guidelines and regulations. All procedures performed in this study were in accordance with the ethical standards of the institutional and/or national research committee and with the 1964 Helsinki Declaration and its later amendments or comparable ethical standards.

### Participants and procedure

This cross-sectional study was conducted between July 15 and 31, 2024. The study targeted a garment production factory with automated equipment. To reduce homogeneity bias, the survey was conducted anonymously. Inclusion criteria were: (1) Currently employed frontline workers. (2) Employees whose production department has undergone or is undergoing digital transformation or technological reform, and who use AI-assisted work and the Fully Automatic Bag Opening Machine daily. (3) At least 1 year of textile work experience, with no history of psychiatric illness or family history of psychosis. The survey was conducted on the “Wenjuan Xing” platform, a professional online survey tool, and distributed via WeChat, a social media platform commonly used by academics for research communication. The survey was administered at a single time point. After obtaining approval from management, participants were informed that the survey was voluntary. The production department has 500 employees, and 400 employees agreed to participate, providing informed consent before answering the questionnaire. To ensure data quality and increase participant engagement, participants were rewarded after completing the survey via the platform. To ensure the quality of the responses, we excluded questionnaires completed in less than 5 min or more than 30 min. Each IP address was allowed only one submission, and responses with inappropriate patterns, such as identical answers to all questions, were discarded.

### Measurement scales

#### Demographic variables

We distributed a self-designed demographic questionnaire to collect participant information, including gender, age, education level (primary, junior, senior, and college and above), and work experience.

#### Challenge–hindrance stressor scale

The challenge-hindrance stressor scale developed by Cavanaugh^11^ includes six items related to challenges and five related to hindrance. This study used the first six questions to determine the pressure of challenge stressors. Responses were collected on a Likert scale, with 1 indicating “very strongly disagree” and 5 indicating “very strongly agree.” An example of a challenge-related item is “The number of projects and assignments I have,” with higher scores indicating high challenge stressors. A previous study has demonstrated the reliability and validity of the challenge–hindrance stressor scale for the Chinese population^15^. The Cronbach’s alpha of the scale in this study was 0.870.

#### Psychological empowerment scale

The psychological empowerment scale developed by Spreitzer^28^ comprises four subscales: meaning, competence, self-determination, and impact, with each scale containing three items each. Responses were collected on a Likert scale, with 1 indicating “very strongly disagree” and 5 indicating “very strongly agree.” Sample items were “The work I do is very important to me” (Meaning), “I am confident about my ability to do my job” (Competence), “I have significant autonomy in determining how I do my job” (Self-determination), and “My impact on what happens in my department is large” (Impact). Spreitzer^28^ explained that these four subfacets additively create psychological empowerment; accordingly, the subscale scores were averaged to obtain a total score for psychological empowerment. A higher score indicated greater perceived empowerment. The reliability and validity of the psychological empowerment scale for the Chinese population has been demonstrated previously^43^. The Cronbach’s alpha of the scale in this study was 0.933.

#### Deviant innovation behavior scale

The deviant innovation behavior scale is based on the scale proposed by Criscuolo^44^, which has five items. Responses were collected on a Likert scale, with 1 indicating “very strongly disagree” and 5 indicating “very strongly agree.” Sample items included “I have the flexibility to work my way around my official work plan, digging into new potentially valuable business opportunities,” with higher scores indicating high deviant innovation behavior. The deviant innovation behavior scale has been demonstrated to be reliable and valid for the Chinese population^33^. The Cronbach’s alpha of the scale in this study was 0.862.

#### Satisfaction with human–robot collaboration scale

The SHRC scale was developed by Cheng and Wu^38^ and is a revision of the satisfaction-related items in Rusbult’s (1998) investment model scale. This scale has six items. Responses were collected on a Likert scale, with 1 indicating “very strongly disagree” and 5 indicating “very strongly agree.” Sample items included, “I think the current collaboration between humans and AI is quite good,” with higher scores indicating high SHRC. The SHRC scale has been demonstrated to be reliable and valid for the Chinese population^38^. The Cronbach’s alpha of the scale in this study was 0.871.

### Statistical analysis

This study used SPSS 27.0 statistical software to analyze reliability and validity and conduct common method bias tests, descriptive statistics, Pearson correlations, and hierarchical multiple regression. The AMOS software was used to conduct confirmatory factor analysis, and a model that included all four variables (i.e., challenge stressors, psychological empowerment, deviant innovation behavior, and SHRC) yielded an acceptable fit: (χ^2^/df = 1.634, RMSEA = 0.045, SRMR = 0.052, CFI = 0.952, TLI = 0.949). All factor loadings were significant (0.58–0.99), demonstrating convergent validity. Values of AVE for each construct were greater than the variance shared with the remaining constructs, suggesting discriminant validity^45^. This study used the Harman single-factor test, and the contribution rate of the first factor was 36.416%, which was less than the standard 40%^46^. This indicates that there is no serious common method bias problem.

## Results

### Sociodemographic characteristics

A total of 400 questionnaires were collected, of which 318 were valid, resulting in a valid return rate of 79.5%. Among the 318 samples obtained, 137 (43.1%) were males and 181 (56.9%) were females. Their ages ranged from 25 to 56 years, with a mean age of 37.06 (SD = 0.51) years. In terms of education level, 57 (17.9%), 102 (32.1%), 87 (27.4%), and 72 (22.6%) participants had completed primary, junior, senior, and college education or above, respectively. Furthermore, 19 (6%), 48 (15.1%), 92 (28.9%), 76 (23.9%), 57 (17.9%), and 26 (8.2%) participants had a work experience of 1, 1–5, 6–10, 11–15, 16–20, and ≥ 21 years, respectively.

### Descriptive statistics and correlation analysis

Pearson correlation analysis was conducted to examine the bivariate correlations among the variables under study. Table 1 shows that challenge stressors positively correlate with deviant innovation behavior (*γ* = 0.601,* p* < 0.01). In contrast, challenge stressors correlate with psychological empowerment (*γ* = 0.478, *p* < 0.01), psychological empowerment positively correlates with deviant innovation behavior (*γ* = 0.576, *p* < 0.01), and SHRC positively correlates with deviant innovation behavior (*γ* = 0.353, *p* < 0.01). The above preliminary data support this study’s main effect and mediating effect hypothesis.

**Table 1:** Results of correlation analysis.

Variable	M	SD	1	2	3	4	5	6	7	8
1. Gender	1.569	0.496	1							
2. Age	3.786	1.904	− 0.064	1						
3. Education	2.547	1.031	0.049	− 0.122*	1					
4. Work experience	3.572	1.327	0.021	0.146**	0.036	1				
5. CS	3.682	0.775	0.030	− 0.035	− 0.027	− 0.026	1			
6. PE	3.663	0.859	− 0.053	0.051	− 0.037	0.008	0.478**	1		
7. SHRC	3.745	0.783	− 0.034	0.114*	− 0.129*	0.012	0.233**	0.308**	1	
8. DIB	2.876	0.870	− 0.039	0.022	0.052	− 0.084	0.601**	0.576**	0.353**	1

Correlation analysis of challenge stressors (CS), psychological empowerment (PE), satisfaction with human–robot collaboration (SHRC), and deviant innovation behavior (DIB) among frontline employees in textile mills (*N* = 318). **p < 0.01 (two-tailed test).

### Hypotheses testing

Table 2 presents the results. For Hypothesis 1, after controlling for gender, age, education level, and years of work experience, the challenge stressor was incorporated into the regression equation alone. It was found to significantly positively predict deviant innovation behavior (Model 4, *β* = 0.610, *p* < 0.001), thus supporting Hypothesis 1. When challenge stressors and psychological empowerment were simultaneously included in the regression equation—after controlling for gender, age, education level, and years of work experience—psychological empowerment was found to significantly positively affect deviant innovation behavior (Model 5, *β* = 0.370, *p* < 0.001). The effect of challenge stressors on deviant innovation behavior decreased compared to Model 2 (*β* = 0.430, *p* < 0.001), indicating that psychological empowerment mediates the relationship between challenge stressors and employee deviant innovation; thus, Hypothesis 2 is verified. To further confirm the mediation effect, a bootstrap analysis with 5,000 resamples was conducted. The indirect effect of challenge stressors on deviant innovation behavior through psychological empowerment was significant (95% CI [0.140, 0.278], excluding 0), providing robust evidence for the mediation effect. Thus, Hypothesis 2 is verified.

**Table 2:** Tests of the mediation effect.

Variable	Psychological empowerment		Deviant innovation behavior		
	Model 1	Model 2	Model 3	Model 4	Model 5
Gender	− 0.05 (0.393)	− 0.06 (0.208)	− 0.04 (0.509)	− 0.06 (0.220)	− 0.03 (0.439)
Age	0.04 (0.446)	0.06 (0.233)	0.04 (0.479)	0.06 (0.181)	0.04 (0.354)
Education	− 0.03 (0.600)	− 0.01 (0.772)	0.06 (0.278)	0.08 (0.074)	0.09 (0.037)*
Work experience	0.01 (0.948)	0.02 (0.784)	− 0.0 (0.111)	− 0.08 (0.084)	− 0.08 (0.044)*
CS		0.48 (0.000)***		0.61 (0.000)***	0.43 (0.000)***
PE					0.37 (0.000)***
R^2^	0.006	0.238	0.013	0.378	0.484
F	0.468	19.472***	1.041	37.939***	48.584***

*p < 0.05; ***p < 0.001.

**Fig. 2:**
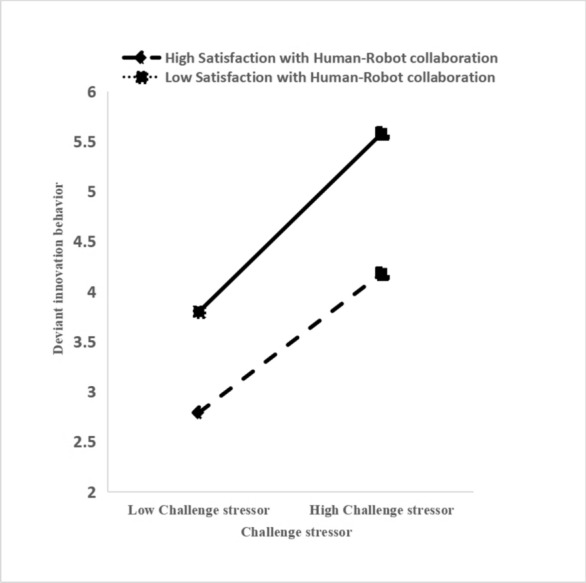
Impact of challenge stressors on deviant innovation behavior under the regulation of satisfaction with human–robot collaboration.

Table 3 shows that when challenge stressors are included in the regression equation alone—after controlling for gender, age, education level, and years of work experience—these stressors can significantly positively predict deviant innovation behavior (Model 2, *β* = 0.610, *p* < 0.001). When SHRC is included in the regression equation—after controlling for gender, age, education level, and years of work experience—SHRC can significantly positively predict deviant innovation behavior (Model 3, *β* = 0.230, *p* < 0.001); the interaction term CS*SHRC also positively predicted deviant innovation behavior (Model 4, *β* = 0.110, *p* < 0.05). Additionally, challenge stressors significantly positively predict deviant innovation behavior at high as well as low SHRC levels, as shown in Fig. 2.

**Table 3:** Tests of the moderation effect.

Variable	Deviant innovation behavior			
	Model 1	Model 2	Model 3	Model 4
Gender	− 0.04 (0.509)	− 0.06 (0.220)	− 0.05 (0.262)	− 0.05 (0.245)
Age	0.04 (0.479)	0.06 (0.181)	0.04 (0.409)	0.04 (0.365)
Education	0.06 (0.278)	0.08 (0.074)	0.11 (0.015)*	0.12 (0.008)**
Year	− 0.09 (0.111)	− 0.08 (0.084)	− 0.08 (0.066)	− 0.08 (0.081)
CS		0.61 (0.000)***	0.55 (0.000)***	0.57 (0.000)***
SHRC			0.23 (0.000)***	0.25 (0.000)***
CS*SHRC				0.11 (0.013)*
R^2^	0.013	0.378	0.428	0.440
F	1.041	37.939***	38.840***	34.751***

* *p* < 0.05; *** *p* < 0.001.

## Discussion

Grounded in the COR theory, this study examines the impact of challenge stressors on frontline employees’ deviant innovation behaviors in Chinese textile mills. It further investigates how psychological empowerment mediates this relationship and how SHRC moderates it. Our findings indicate that challenge stressors positively influence deviant innovation behavior, with psychological empowerment acting as a mediator and SHRC as a moderator. And this study examines the role of AI and automation in mediating stress and facilitating employee deviant innovation, addressing a gap in the existing literature.

First, we find that challenge stressors significantly enhance the deviant innovation behavior of frontline workers in textile mills, confirming the relevance of COR theory. Challenge stressors are common among these employees, yet previous research has primarily focused on the sources and consequences of stress^17,24^. The mechanisms by which employees respond to these pressures, particularly regarding unverified methods or challenging existing processes, remain underexplored. Unlike hindrance stressors, challenge stressors can stimulate intrinsic motivations such as self-efficacy, work meaning, and competence. They promote employees’ motivation, fostering personal growth and career development^11,17,19^. Our study confirms that employees perceive challenge stressors as opportunities to leverage resources like knowledge, skills, and organizational support. This perspective not only enhances their coping abilities but also fosters a positive attitude toward stress, driving continuous deviant innovation.

Second, we demonstrate that psychological empowerment mediates the relationship between challenge stressors and deviant innovation behavior among frontline workers, validating the roles of cognitive appraisal and self-determination theories. While previous studies have addressed factors like job satisfaction and self-efficacy in relation to innovation, they primarily focus on promoting general innovation capabilities. In contrast, deviant innovation behavior, characterized by higher risk and uncertainty^18^, requires individuals to be willing to address challenges and seize opportunities despite potential costs^47^. Thus, employees’ perceptions of their organizational environment significantly influence their willingness to engage in deviant innovation. Psychological empowerment facilitates this behavior by allowing employees to assess risks and outcomes confidently. With high psychological empowerment, employees can pursue deviant innovation without excessive concern for risks, leading to bolder and more enthusiastic attempts at new ideas. Our study highlights that challenge stressors not only stimulate innovation but also serve as prerequisites for psychological empowerment, expanding its applicability and revealing its role in fostering high-risk innovation.

Thirdly, we extend situational awareness theory by examining SHRC’s moderating role in the relationship between challenge stressors and deviant innovation behavior among frontline textile workers in China. High SHRC amplifies the positive effects of challenge stressors on deviant innovation behavior. The rapid advancement of technologies, particularly AI and automation, has made human–robot collaboration prevalent in many workplaces^1,4^. When employees are satisfied with their collaboration with advanced technology, they feel empowered to manage routine tasks effectively, freeing time and energy for innovation. This mechanism elucidates how technology and human–robot collaboration can indirectly promote deviant innovation behavior. While prior studies have linked technology to innovation, few have explored how satisfaction with technology impacts deviant innovation behavior. High SHRC enhances employees’ self-efficacy and enthusiasm for deviant innovation, providing insights into how technological support and human–robot collaboration can stimulate innovation potential.

Finally, theoretically, this study contributes to COR theory by demonstrating how challenge stressors can enhance deviant innovation through resource optimization and psychological empowerment, expanding our understanding of stress’s dual role in workplace innovation. Moreover, the integration of situational awareness theory highlights SHRC as a crucial moderator, bridging gaps in existing literature regarding the relationship between human–robot collaboration and employee innovation^1,17,19^. This research also emphasizes the role of psychological empowerment in fostering high-risk innovation, offering new insights into self-determination and cognitive appraisal theories. Practically, the findings suggest that organizations should cultivate a supportive environment that reframes challenge stressors as opportunities, empowering employees through adequate resources, training, and AI-enabled tools. Enhancing SHRC can provide employees with confidence and motivation to innovate, particularly in high-stress, technology-driven workplaces. Managers should address barriers to innovation, such as skepticism about AI, through clear communication and capacity-building initiatives, ultimately fostering a culture that balances innovation with risk management^24,47^. Furthermore, this study’s findings offer practical insights for textile factories and other similar industries, especially during technological transformations. By recognizing challenge stressors as opportunities for growth and innovation, organizations can implement strategies that foster a culture of innovation, even under pressure. In particular, the integration of AI and automation into production lines can serve as a catalyst for creative problem-solving. For textile factories, this research suggests that management should provide employees with the tools, training, and support they need to navigate technological changes, thereby enhancing both productivity and employee satisfaction. While this study was conducted in a single textile factory, the underlying mechanisms of challenge stressors, psychological empowerment, and SHRC can be generalized to other manufacturing sectors undergoing digital transformation. Future research should examine the applicability of these findings across diverse industries to assess the robustness of the proposed model. It is also important to explore how industry-specific factors, such as the level of technological adoption and organizational culture, may influence the relationship between stressors and innovation behaviors. By investigating a broader range of industries, researchers can better understand the generalizability of these findings and refine strategies for fostering innovation in varying contexts.

### Limitations and implications

This study relies on cross-sectional data, preventing the determination of causal relationships between variables. Future research should assess the stability of this theoretical model (CS-PE-DIB) across various industries. A longitudinal approach could clarify the causal link between challenge stressors and deviant innovation, enhancing the precision of the findings.

Second, this study does not fully address the deviant innovation behavior of employees from diverse cultural backgrounds. In China’s conservative and hierarchical context, employees may exhibit limited deviant innovation due to caution and reluctance to challenge the status quo. Future research should explore how Chinese organizations can adapt management strategies to foster innovation while maintaining stability.

Third, this study focuses solely on the mechanisms through which challenge stressors influence deviant innovation behavior. It does not consider the potential benefits and drawbacks of deviant innovation or the impact of other influencing factors.

Future research could explore the role of different dimensions of psychological empowerment in the relationship between challenge-hindrance stressors and deviant innovation behavior in frontline employees. And future research could investigate the risks associated with deviant innovation and how organizations can alleviate employee concerns through effective risk management and support systems. This approach would aid in developing resources to support employee innovation and establish a coherent framework for studying the impact of challenge stressors.

Lastly, regarding the generalizability of the findings to other companies, it is important to acknowledge that this study was conducted in a single textile factory. While the mechanisms identified may be relevant to other industries undergoing similar technological transformations, the specific organizational culture and contextual factors in this study limit its applicability to other sectors. Future research should examine how industry-specific characteristics, such as the degree of technological adoption, organizational structure, and cultural differences, may influence the relationship between challenge stressors and deviant innovation. This would provide a clearer understanding of how the model can be applied across different contexts.

## Conclusion

This study examines the interaction between external factors (challenge stressors), individual perceptions (psychological empowerment and SHRC), and behavioral responses (deviant innovation behavior), focusing on how challenge stressors influence deviant innovation. Utilizing the COR theory, we analyze the direct impact of challenge stressors on deviant innovation behavior. The indirect effects of psychological empowerment are explored through cognitive appraisal and self-determination theories, while SHRC’s indirect influence is analyzed through the lens of situational awareness theory.

The main conclusions are as follows: First, challenge stressors have a significant positive effect on the deviant innovation behavior of frontline employees. Second, psychological empowerment mediates the relationship between challenge stressors and deviant innovation behavior. Finally, SHRC moderates this relationship, with the positive impact of challenge stressors on deviant innovation behavior increasing when SHRC is high. This study contributes to empirical research in human resources, offering insights for enhancing stress management and promoting deviant innovation among frontline employees.

## Data Availability

Due to privacy, the datasets from this study are not publicly available but can be requested from the corresponding author.
